# Dissection of the catalytic and regulatory structure-function relationships of Csk protein tyrosine kinase

**DOI:** 10.3389/fcell.2023.1148352

**Published:** 2023-03-01

**Authors:** Gongqin Sun, Marina K. Ayrapetov

**Affiliations:** Department of Cell and Molecular Biology, University of Rhode Island, Kingston, RI, United States

**Keywords:** Csk and Chk, Src regulation, substrate recognition, kinase regulation, structure-function relationships, domain-domain interaction, redox regulation

## Abstract

Protein tyrosine kinases (PTKs) are a large enzyme family that regulates many cellular processes. The key to their broad role in signaling is their tunable substrate specificity and regulatory mechanisms that allow each to respond to appropriate regulatory signals and phosphorylate the correct physiological protein substrates. Thus, in addition to the general PTK catalytic platform, each PTK acquires unique structural motifs that confer a unique combination of catalytic and regulatory properties. Understanding the structural basis for these properties is essential for understanding and manipulating the PTK-based signaling networks in normal and cancer cells. C-terminal Src kinase (Csk) and its homolog, Csk-homologous kinase (Chk), phosphorylate Src family kinases on a C-terminal Tyr residue and negatively regulate their kinase activity. While this regulatory function is biologically essential, Csk and Chk have also been excellent model PTKs for dissecting the structural basis of PTK catalysis and regulation. In this article, we review the structure-function studies of Csk and Chk that shed light on the regulatory and catalytic mechanisms of protein tyrosine kinases in general.

## 1 Introduction

The function of Csk was understood long before the Csk gene or protein was identified. In the late 1970s, viral Src (v-Src) was established as the causative agent of oncogenic transformation in the Rous Sarcoma Virus (Coffin, 1976; Kawai et al., 1977; Purchio et al., 1978), while its non-transforming counterpart, cellular Src (c-Src) gene, was found to be part of the mammalian and avian genomes (Oppermann et al., 1979; Shalloway et al., 1981). A comparison of amino acid sequences between v-Src and c-Src revealed that their main difference is in the C-terminal tail: c-Src contains a C-terminal tail absent in v-Src (Takeya & Hanafusa, 1983). This comparison suggested that the C-terminal tail suppresses the transforming potential of c-Src. It contains a Tyr residue (Tyr530 in human c-Src) that is phosphorylated (Cooper et al., 1986). This phosphorylation suppresses the c-Src kinase activity and its transforming potential (Cooper & King, 1986; Cooper & Runge, 1987). The phosphorylation of the C-terminal tail Tyr was catalyzed by a different protein tyrosine kinase, instead of by Src autophosphorylation (Thomas et al., 1991). This kinase, named C-terminal Src kinase (Csk), was identified (Okada & Nakagawa, 1988; 1989), purified (Okada et al., 1991), and its gene was cloned (Nada et al., 1991; Okada et al., 1991). A Csk-homologous kinase (Chk) was independently identified and cloned by several research groups (Bennett et al., 1994; Kuo et al., 1994; McVicar et al., 1994; Sakano et al., 1994). All eight members of the human Src family kinases (SFKs), Src, Yes, Fyn, Lck, Lyn, Hck, Fgr and Blk, are regulated by Csk and Chk in a similar manner (Parsons & Parsons, 2004). Csk and Chk defined a novel family of PTKs with distinct substrate specificity, catalytic and regulatory characteristics, and structural organization.

• Csk’s role in Src regulation and the ease of recombinant expression and purification in a bacterial system positioned Csk as an excellent model enzyme for studying the structure-function relationships in PTK catalysis and regulation. This article reviews the studies contributing to our current understanding of PTK biochemistry.

## 2 General structural features of Csk and Src

**FIGURE 1:**
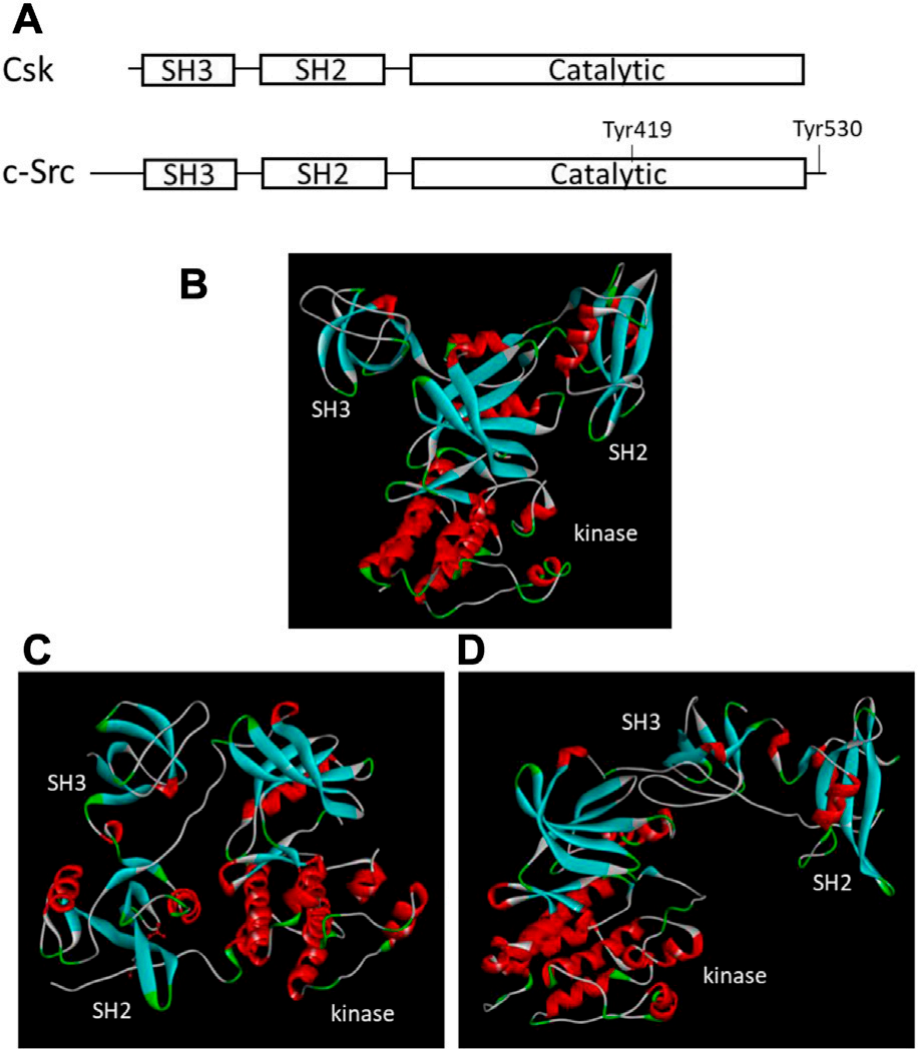
Structural organizations and tertiary structures of Csk and Src. **(A)**. Domain organization of Csk and Src. The key domains and Tyr residues important for regulation are indicated. **(B)**. Tertiary structure of Csk (PDB ID 1k9A, Ogawa et al., 2002). **(C)**. Tertiary structure of Src when Tyr530 is phosphorylated (PDB ID 1FMK, Xu et al., 1999). **(D)**. Tertiary structure of Src when Tyr530 is unphosphorylated (PDB ID 1Y57, Cowan-Jacob et al., 2005). The structural images are generated using Discovery Studio Visualizer.

Since Csk was identified first, the enzyme attracted most of the research attention and became the Csk family representative by default. It is comprised of 450 amino acids organized into several distinct domains conserved in many PTKs and other signaling proteins. Csk shares a 54% sequence identity with Chk and 40% with Src. All three enzymes have a similar SH3-SH2-catalytic domain organization (Figure 1A). However, Csk lacks several structural features found in Src family kinases: the ∼80-residue unique domain at the N-terminal region, an autophosphorylation site in the autophosphorylation loop or activation loop, and the C-terminal tail containing a Tyr residue. Because these structural features play essential roles in the regulation of Src, their absence suggests unique regulatory strategies for Csk.

The crystal structures of the Csk (Ogawa et al., 2002) and Src (Xu et al., 1999; Cowan-Jacob et al., 2005) also show a different tertiary arrangement of the SH3 and SH2 domains relative to the kinase domain (Figure 1). In Csk, the SH3 and SH2 domains directly interact with the small lobe of the kinase domain. This domain arrangement is not regulated by phosphorylation or other regulatory events. The domain arrangement in Src is regulated by C-terminal Tyr phosphorylation. Phosphorylated Tyr530 binds to the SH2 domain, changes the domain arrangement in Figure 1C, and shifts Src into an inactive conformation (Cooper & Howell, 1993; Xu et al., 1999). When Tyr530 is not phosphorylated, Src is active and adopts a different domain arrangement, as shown in Figure 1D (Cowan-Jacob et al., 2005).

## 3 Csk and Src have distinctive substrate specificity and regulatory strategies

Despite many structural similarities, Csk and Src family kinases have highly distinct substrate specificity and regulatory characteristics. Csk has an unusually stringent substrate specificity, while Src is known to phosphorylate many protein substrates (Brown & Cooper, 1996). Csk and Src play opposing roles in Src regulation due to their contrasting substrate specificity. Src is activated by trans-molecular autophosphorylation on Tyr419 in the activation loop (Sicheri & Kuriyan, 1997; Sun et al., 1998) and inactivated by the phosphorylation of Tyr530 by Csk (Okada et al., 1991; Okada, 2012). Furthermore, autophosphorylation-induced activation overrides the inactivation by Tyr530 phosphorylation (Sun et al., 1998). Thus, Src recognizes and autophosphorylates Tyr-419, while Csk recognizes and phosphorylates Src on Tyr530. The distinct substrate specificity of Csk and Src in recognizing and phosphorylating the correct Tyr residues on Src is essential for proper Src regulation. These phosphorylation events represent a well-defined model system for PTK substrate specificity.

The contrast between the regulatory mechanisms for Src and Csk is also quite striking. The Src kinase domain alone is intrinsically active. This activity is suppressed by the interactions between the kinase and the regulatory domains (Weijland et al., 1996; Seeliger et al., 2005). The binding of pTyr530 to the SH2 domain induces a series of domain-domain interactions, which result in Src inactivation (Cooper & Howell, 1993; Xu et al., 1999). Other regulatory mechanisms are primarily based on ways to control these interactions (Bjorge et al., 2000). For example, dephosphorylation of pTyr530, ligand binding to the SH2 domain and/or the SH3 domain, or autophosphorylation can all disrupt this inactive conformation and lead to Src activation (Bjorge et al., 2000). In contrast, the Csk kinase domain is intrinsically inactive (Sondhi & Cole, 1999) and is activated by interactions with the regulatory SH3 and SH2 domains. It is not regulated by Tyr phosphorylation and lacks a phosphorylation site in the activation loop, even though the conformation of the activation loop does control the kinase activity of Csk (Lin et al., 2003; Levinson et al., 2008).

The contrasting substrate specificity and regulatory strategies embedded in the two highly homologous kinase structures of Csk and Src make these enzymes particularly attractive for structure-function studies. Csk is also a unique PTK because it can be readily expressed in bacteria without causing significant cellular toxicity (Bougeret et al., 1993). This is likely due to its strict substrate specificity, as the expression of other PTKs, such as Src, is toxic to bacteria (Kemble et al., 2006; Wang et al., 2006).

## 4 Csk as a model for investigating PTK catalysis and inhibitor design

Csk has served as a model for studying the catalytic mechanisms of PTKs. Cole and others have extensively probed Csk’s catalytic mechanism using a combination of enzyme mutagenesis (Cole et al., 1995; Williams et al., 2000; Williams & Cole, 2002), protein ligation (Muir et al., 1998), unnatural substrates (Sondhi et al., 1998; Kim et al., 2000; Wang & Cole, 2001), nucleotide-peptide substrate linkage (Shen & Cole, 2003; Hines & Cole, 2004), and chemical probing (Cole et al., 1994; Grace et al., 1997; Williams et al., 2000; Muratore et al., 2009). These studies have been previously reviewed (Cole et al., 1999; Cole et al., 2003; Shen et al., 2005; Wang & Cole, 2014). These efforts defined the roles of Csk catalytic base, Asp314, and metal cations in PTK catalysis, revealed a random order of substrate binding mechanism, and demonstrated a dissociative transition state mechanism for phosphoryl transfer. These mechanistic understandings have led to the development of bi-substrate inhibitors for PTKs (Parang et al., 2001; Parang & Cole, 2002; Hines et al., 2005) and the strategy of using chemical rescue to activate kinase-defective mutants and help identify physiological substrates for PTKs (Qiao et al., 2006; Ferrando et al., 2012). Although these studies started with Csk, their findings have been extended and applied to other PTK families.

Divalent metal ions have two positive charges and can coordinate with multiple Lewis base functional groups in protein structure and enzyme catalysis. These unique properties enable divalent metal cations to strongly influence electron redistribution in chemical reactions and play broad and essential roles in enzyme catalysis (Andreini et al., 2008). PTKs also use divalent metal cations to aid the phosphoryl transfer reaction. Cole and coworkers noted that either Mn^2+^ or Mg^2+^ ion could support Csk catalytic activity, with a Mn^2+^ ion resulting in a more favorable K_m_s for ATP and peptide substrates, while a Mg^2+^ ion results in a more favorable k_cat_ (Grace et al., 1997). Sun and Budde further defined the role of divalent metal cations in Csk catalysis (Sun & Budde, 1997). They found that Csk and other PTKs use two kinetically definable divalent metal cations to catalyze the phosphoryl transfer reaction: one bound to ATP to form the ATP-metal complex as the substrate and the other bound to the active site to serve as a catalytically essential activator. The second metal ion promotes K_cat_ without affecting substrate binding (the ATP-metal complex or the peptide) in Csk. The conclusion was also extended to other PTKs, such as Src and FGFR1 (Sun & Budde, 1997). The crystal structure of insulin receptor kinase confirmed the presence of two divalent metal cations in the catalytic complex (Hubbard, 1997).

Although Mg^2+^ is generally considered the physiological metal ion in PTK catalysis due to its availability (Maguire & Cowan, 2002), either Mn^2+^, Ni^2+^, or Co^2+^ can also act as the essential activator, with Mn^2+^ being the most effective (Sun & Budde, 1999b). Surprisingly, Zn^2+^ binds to the second metal binding site with 13,200-fold higher affinity than Mg^2+^. However, instead of supporting Csk catalytic activity, it acts as a potent Csk inhibitor (Sun & Budde, 1999b). The binding of Zn^2+^ to Csk is so tight that it can be used for immobilized metal affinity purification of Csk from bacterial lysate overexpressing it (Sun & Budde, 2001). The finding of the second metal binding site also resulted in the development of metal-mediated inhibitors for Csk in Co^2+^-amino acid hydroxamate complexes (Gu et al., 2006).

In the cellular context, Mg^2+^ is likely the metal ion in Csk and other PTK catalysis because of its high concentration in the cytosol (Maguire & Cowan, 2002). Furthermore, growth factors, such as insulin (Sanui & Rubin, 1978; Hwang et al., 1993) and epidermal growth factor (Grubbs, 1991), also stimulate cellular Mg^2+^ uptake and increase cellular free Mg^2+^ concentration. Thus, PTK activation due to increased Mg^2+^ concentration could be a global response to the growth factor stimulation.

## 5 Csk as a model for PTK substrate recognition

Over 500 protein kinases are encoded by the human genome, including more than 90 PTKs (Manning et al., 2002). The kinome regulates over 30% of all cellular proteins, and substrate specificity is crucial for the kinome-based signaling. The substrate specificity of PTKs determines the specificity and fidelity of the protein tyrosine phosphorylation-based regulatory system. While some PTKs, such as Src, seem to have a broad substrate specificity (Brown & Cooper, 1996), others, such as Csk, phosphorylate only a very small number of substrates. The mechanism of PTK substrate recognition and specificity has attracted considerable attention in the kinase field. Because Csk has a strict and well-defined substrate specificity, it served as an excellent model for elucidating the mechanism of PTK substrate recognition.

Substrate recognition by Ser/Thr protein kinases tended to be based on the peptide sequence surrounding the phosphorylation sites (Kemp & Pearson, 1990). Initial efforts investigating PTK substrate specificity also focused on the peptide substrates (Budde et al., 1995; Ruzzene et al., 1997). However, it soon became apparent that Csk recognition of Src Tyr530 is not based on the local peptide sequence surrounding Tyr530. Similar optimal peptide substrates for Csk have been independently identified (Sondhi et al., 1998; Deng et al., 2014); however, the peptide sequences have no similarity to the C-terminal tail sequence of Src family kinases (Sondhi et al., 1998). Sondhi et al. (Sondhi et al., 1998) further developed a kinase-defective Src (kdSrc, due to K295M mutation) as an efficient substrate for Csk and dissected the contribution of various domains of Csk and Src to this Csk-Src recognition (Wang et al., 2001). The study concluded that the recognition was achieved by tertiary structural interactions between the catalytic domains of the two kinases as well as the interaction between the Csk catalytic domain with some residues near the phosphorylation site (Wang et al., 2001).

**FIGURE 2:**
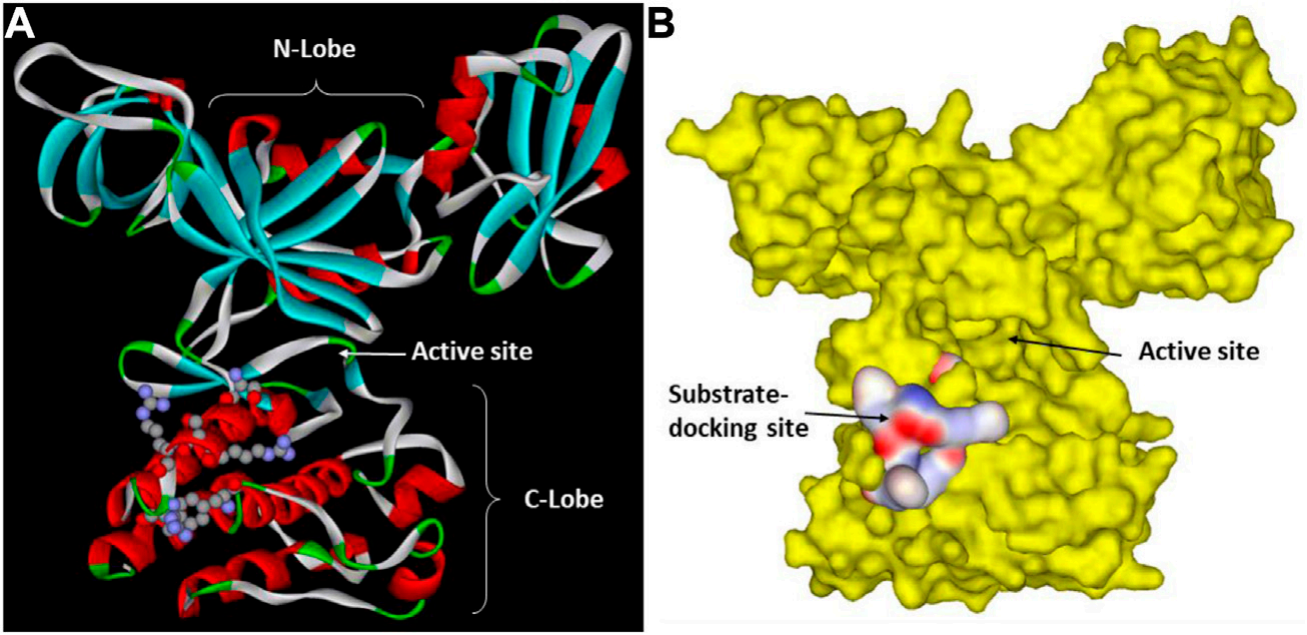
Csk substrate docking site. **(A)**. Csk ribbon structure with the key residues in the substrate docking site shown in ball and stick. The N-lobe, C-lobe and the active site cleft of Csk are indicated to show their positions relative to the substrate-docking site. **(B)**. Surface structure of Csk and the substrate-docking site colored by the electric potential.

Based on these insights and using the kdSrc substrate, Lee et al. mapped the substrate-docking site on the Csk catalytic domain responsible for recognizing Src (Lee et al., 2003; Lee et al., 2006). One key clue came from the observation that a relatively small number of residues are uniquely conserved between Csk and Chk but not in other PTK catalytic domains. They reasoned that these residues are likely involved in some function unique to Csk and Chk, presumably substrate recognition. This indeed was the case as a group of residues uniquely conserved in Csk and Chk were specifically important for Csk’s ability to phosphorylate kdSrc but not crucial for its phosphorylation of an artificial peptide substrate, polyE_4_Y. Mutation of these residues, Arg279, Ser280, Arg281, Arg283, and Phe382, specifically abolished Csk activity toward its physiological substrate but not the artificial substrate. These residues are located in a cluster on the peptide-binding lobe of Csk (Lee et al., 2003). The substrate-docking site of Csk relative to the active site is illustrated in Figure 2.

Lee et al. further mapped the residues on Src that are recognized by the Csk substrate-docking site (Lee et al., 2006). They demonstrated that even when all six residues surrounding Tyr530, three on each side, were mutated to Ala, kdSrc can still serve as an efficient Csk substrate. Using Ala-scanning, they identified three key Src residues, Glu510, Tyr511, and Asp518, essential for Csk recognition of kdSrc. Mutation of all three residues to Ala decreased kdSrc’s ability to serve as a substrate by 98.6% from 258 s^-1^ to 3.6 s^-1^. Furthermore, these residues are conserved in Src family kinases. Mutation of the equivalent residues in Yes, another Src family kinase, did not directly affect Yes kinase activity but abolished Yes’ ability to be phosphorylated and inactivated by Csk (Lee et al., 2006). They also demonstrated that Glu510 of Src directly interacts with Arg283 of Csk, a key residue in the Csk substrate-docking site.

While these mutational studies identified some of the residues in Csk and Src that mediate the kinase-substrate interaction, the most definitive evidence for this docking-based substrate recognition was obtained from the crystal structure of the Csk and Src complex (Levinson et al., 2008). The crystal structure of the Csk and Src complex confirmed the docking interactions between Csk and Src that position the Src C-terminal tail at the edge of the active site of Csk. This study also revealed that the shorter-than-usual activation loop of Csk also contributes to the extraordinary substrate specificity of Csk in only phosphorylating Src family kinases using the tertiary structural interaction mechanism (Levinson et al., 2008). To our knowledge, this remains the only PTK-substrate system where the mechanism of recognition was structurally defined. It remains a question if a similar mechanism of substrate recognition is adopted by other PTKs, even though it is demonstrated that Src also uses its C-lobe to recruit protein substrates (Wang et al., 2007).

While the direct interactions between Csk and Src initiate Csk substrate recognition, kinetic mechanism is also a major factor in determining Csk substrate specificity. Adams and others used pre-steady-state and transient-state kinetics to investigate the contribution of substrate binding, phosphoryl transfer, and product release to Csk-Src recognition (Shaffer et al., 2001; Lieser et al., 2005a; Lieser et al., 2005b; Lieser et al., 2006). They demonstrated that the physical interaction between Csk and Src is weak (larger *K*_*d*_), but the catalytic affinity is much stronger (much lower *K*_*m*_). The low *K*_*m*_ is due to the fast phosphoryl transfer that acts as a kinetic clamp facilitating substrate recognition (Lieser et al., 2005a; Lieser et al., 2005b). They determined that substrate association, phosphoryl transfer and product release are fast and non-rate-limiting, and a Csk conformational change during the catalytic cycle acts as the limiting step for the turnover. These studies provide a more complete and dynamic picture to Csk substrate recognition and phosphorylation of Src family kinases.

In addition to direct interaction between Csk and the SFKs, an adaptor protein, which recruits Csk and an SFK to the same cellular location, may also play a major role in facilitating Csk phosphorylation of SFKs (Tawaratsumida et al., 2022). Interferon regulatory transcription factors (IRFs) orchestrate key effector functions, including cytokine release, cell differentiation, and, under certain circumstances, inflammation pathology. Tawaratsumida et al. demonstrated that the proteasomal degradation of IRFs is controlled *via* phosphorylation of a conserved Tyr residue by the Src family kinase Lyn. The activity of Lyn is inactivated by Csk *via* phosphorylation of the C-terminal Tyr residue. The ability of Csk to phosphorylate Lyn requires the adaptor protein speckle-type POZ protein (SPOP), which recruits Csk to the IRF signaling complex and activates Csk activity. They further identified the SPOP-binding motifs in the Csk activation loop. It is unclear if and how the adaptor protein may alter the physical and catalytic interaction between Csk and Lyn in this process besides bringing them to the same complex. Although SFKs are the most well recognized substrates for Csk, other protein substrates have been identified for Csk. D’Arco et al. identified ATP-activated P2X_3_ receptor as a Csk substrate (D'Arco et al., 2009) and demonstrated that Csk-mediated Tyr393 phosphorylation regulates P2X3 receptor function in mouse sensory neurons. Yao et al. (Yao et al., 2014) demonstrated that Csk phosphorylates eukaryotic elongation factor 2 (eEF2) on Tyr265 and Tyr373 and regulates its cleavage and cellular localization. While these studies further expand the physiological roles of Csk, they also pose interesting questions about Csk and PTK substrate recognition. Since the structures of these substrates have little in common with those of SFKs, these studies make it clear that Csk can recognize SFKs and other unrelated protein substrates. It would be interesting to determine if Csk uses the same or a different substrate docking site for recognizing these other substrates, and if and how such substrate recognition sites are related to the non-catalytic but inhibitory interaction between Chk and SFKs (Advani et al., 2017). Furthermore, Csk also has preferred peptide substrates (Sondhi et al., 1998; Deng et al., 2014). Thus, multiple motifs related to substrate recognition are likely embedded in the Csk/Chk catalytic domains. Elucidating these recognition mechanisms may shed light on the even broader question of how PTKs with broad substrate specificity, such as Src, recognize many unrelated protein substrates.

## 6 Csk regulation by domain-domain interaction

 Although Csk and Src share a 40% amino acid identity and similarity in domain organization, early studies indicated that these two kinases have different regulatory strategies. The isolated Src kinase domain is active and inhibited by its interactions with the regulatory domains (Cooper & Howell, 1993). Various mechanisms of Src regulation are based mainly on manipulating this basic mechanism (Bjorge et al., 2000). However, Csk regulation uses a different strategy. The kinase domain of Csk is intrinsically inactive, and the regulatory domains in Csk stimulate its catalytic activity (Sabe et al., 1994; Cloutier et al., 1995; Koegl et al., 1995). Mutational studies indicated that deletion and mutation to the regulatory domains result in a loss of over 90% of the Csk catalytic activity (Sun & Budde, 1999a; Sondhi & Cole, 1999). Sondhi and others further demonstrated that the isolated Csk catalytic domain is mainly inactive and can be activated by incubation with Csk SH3 and SH2 domain fragments (Sondhi & Cole, 1999). These results demonstrated that the Csk kinase domain is intrinsically largely inactive, and the regulatory domains activate it through domain-domain interactions.

 Using NMR and site-specific mutagenesis, Shekhtman et al. demonstrated that the Csk SH3 domain and the SH3-SH2 linker play essential roles in interacting with and stimulating the activity of the Csk kinase domain (Shekhtman et al., 2001). The crystal structure of full-length Csk (Ogawa et al., 2002) confirmed the interactions between the regulatory domains with the kinase domain. It revealed a structure with an arrangement of SH3, SH2 and kinase domain distinctly different from that of Src (Xu et al., 1999) (Figure 1). The crystal structure provided a framework for further defining the regulatory mechanisms of Csk.

Using Csk tertiary structure as a guide, Lin et al. dissected the structural basis for the domain-domain communication in Csk (Lin et al., 2006). They showed that the regulatory domains are important in activating the catalytic domain, but they are not involved in recognizing the physiological substrate, Src (Lin et al., 2005). They identified a Cation-π interaction between Arg68 from the SH3-SH2 linker and Trp188 from the kinase domain that is crucial for the SH3 domain activation of Csk, as mutation of Arg68 to Ala decreased Csk activity >10-fold, equivalent to the deletion of the entire SH3 domain.

The SH3 domain of Csk binds to the Pro-rich motifs of protein tyrosine phosphatases PTPN12 (PTP-PEST) (Davidson et al., 1997; Ghose et al., 2001) and immune-cell PTPN22 (LYP/Pep) (Cloutier & Veillette, 1996; Gregorieff et al., 1998). This binding does not directly affect Csk catalytic activity (Sondhi & Cole, 1999), but recruits the phosphatases that dephosphorylate the SFK activation loop. Because SFK inactivation requires the dephosphorylation of the activation loop Tyr and the phosphorylation of the C-terminal tail Tyr, the Csk-phosphatase complex can more effectively inactivate SFKs. Interestingly, Csk can also homodimerize through its SH3 domain *in vitro* (Levinson et al., 2009) and in Jurkat T-cell (Brian et al., 2022), and the dimerization precludes the binding of the Csk to the phosphatases. Mutations in the Csk SH3 domain can selectively disrupt the dimerization or phosphatase binding, indicating overlapping but not identical interfaces for the two types of interactions (Brian et al., 2022). These studies suggest a more complex regulation of Csk function by protein-protein interactions that can manipulate the basic catalytic function of Csk.

The Csk SH2 domain binds to a pTyr residue in the Csk binding protein, CBP, which serves two roles: recruiting Csk to the lipid rafts where SFKs are located (Yasuda et al., 2002; Xu et al., 2005; Matsubara et al., 2010; Kanou et al., 2011) and directly activating Csk (Kawabuchi et al., 2000; Takeuchi et al., 2000; Lin et al., 2005; Matsubara et al., 2010). Thus, the binding to CBP greatly enhances Csk function in SFK regulation. In non-small cell lung cancer (NSCLC) cell lines that have upregulated c-Src, CBP expression is significantly downregulated. The ectopic expression of CBP suppresses the anchorage-independent growth of the NSCLC cell lines (A549 and Lu99) and suppressed the kinase activity of c-Src by recruiting it and Csk to lipid rafts (Kanou et al., 2011). A similar recruiting mechanism is observed in numerous other cell types, such as T-cell (Yasuda et al., 2002; Xu et al., 2005), osteoclasts (Matsubara et al., 2010), and spleen macrophages (Matsubara et al., 2010).

The Csk SH2 domain activates Csk activity constitutively and also in a ligand-binding-dependent manner. To understand the structural basis of the SH2-mediated regulation, Lin et al. dissected the functional roles of the residues in the SH2 and catalytic domain interface by site-specific mutagenesis and kinetic analyses (Lin et al., 2006). They identified three functionally distinct types of residues mediating the communication between the SH2 and the catalytic domains. Type I residues mediate a ligand-triggered activation of Csk, because their mutation severely reduces Csk activation by the SH2 domain ligand. Type II residues are involved in suppressing Csk activity. Their mutation activates Csk but makes Csk less sensitive to activation by the SH2 ligand. Type III residues are located in the SH2 domain and activate Csk kinase activity remotely. Their mutation severely decreases Csk catalytic activity without affecting the SH2 ligand-triggered activation. These residues likely mediate SH2 activation of Csk regardless of SH2-ligand interaction.

**FIGURE 3:**
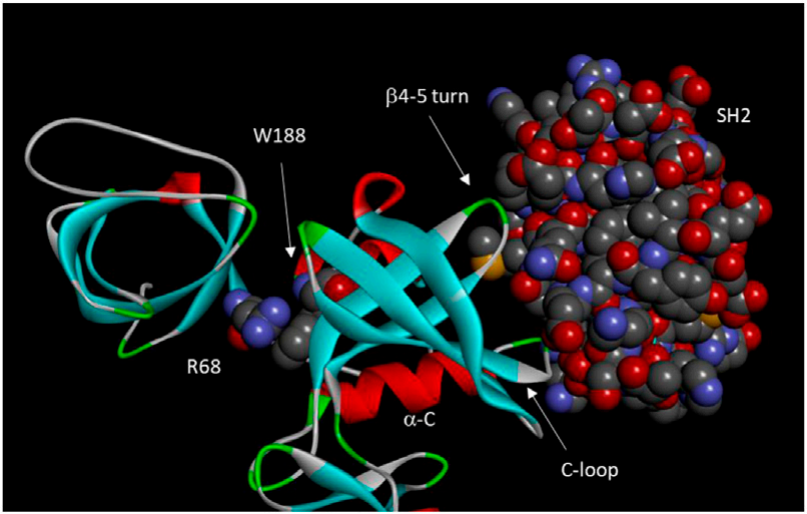
Domain-domain interactions in Csk. Important residues and motifs are indicated. Arg68, Trp 188, and the Csk SH2 domain are shown in space-filling model. The motifs of a-C, C-loop, and b4-5 turn constitutes an “on switch” in Src kinase domain. However, these motifs significantly diverged from those in Src to become a defective “on switch” that can only be turned on by interactions with the SH2 domain.

These studies suggest a two-tiered regulatory model of Csk by the regulatory domains. First, the catalytic domain is intrinsically inactive and activated by interactions with the SH3 domain (such as the cation-π interaction between Arg68 and Trp188) and the SH2 domains (SH2 domain residues Met150 and Asn148). Second, some SH2 domain residues (Glu154 and Glu258) also suppress the catalytic activity, keeping the catalytic activity suboptimal. This suppression is removed by the SH2 domain binding to a ligand. Several residues involved in the SH2 ligand-stimulated activation are D227, T229, Y133, and E147. Critical residues and motifs for Csk domain-domain interactions are shown in Figure 3.

At the heart of the divergent regulatory strategies in Csk and Src kinases is this question: why is the Csk catalytic domain intrinsically inactive while the Src catalytic domain is intrinsically active? The answer to this question may also shed light on the regulatory strategies of eukaryotic protein kinases in general. Some kinases are like Csk and have an intrinsically inactive catalytic domain that is activated by the regulatory domain or subunits (such as cyclin-dependent protein kinases (Wood & Endicott, 2018)), while others are like Src and have intrinsically active kinase domains that are suppressed by regulatory domains or subunits (such as cAMP-dependent protein kinase (Taylor et al., 1990)). Using Csk-Src chimeras switching various subdomains, it was shown that the regulatory strategies are determined by the small lobe (N-lobe), and the substrate specificity is determined by the large lobe (C-lobe) of the catalytic domain (Wang et al., 2007).

7. Huang et al. further analyzed the structural basis of the opposing regulatory strategies in Csk and Src. They identified structural elements in the N-terminal lobe of the catalytic domain that render the Src kinase domain intrinsically active (Huang et al., 2009). These structural elements include the α-helix C region, a β-turn between the β4 and β5 strands, and an Arg residue at the beginning of the catalytic domain. These three motifs interact with each other to keep the Src catalytic domain active. Thus, these motifs and their interactions act as the “on switch” in Src. The equivalent motifs in Csk significantly diverged from those in Src. Thus, the “on switch” is missing in Csk. However, these equivalent motifs in Csk directly interact with the Csk SH2 domain, which activates the Csk kinase domain. Strikingly, the Src “on switch” motifs can be grafted to the Csk kinase domain to obtain an intrinsically active Csk kinase domain. These results revealed that the Src N-lobe contains a structural “on switch”, but Csk has a defective “on switch”, which can be turned on by interactions with the SH2 domain. In both Csk and Src, the regulatory strategies are largely built on manipulating these “on switches”. While site-specific mutagenesis coupled to kinetic analysis can identify key structural motifs involved in Csk regulation, the combination of kinetic analysis, molecular dynamics simulations, and deuterium exchange mass spectrometry provided a much better understanding of the conformational coupling between the regulatory and the catalytic domain in Csk. Jennings and others demonstrated that the conformations of the SH2, SH3 domains and the catalytic domain are dynamically coupled. The domain–domain interactions, controlled through the SH2-kinase linker, provide a dynamic balance within the Csk framework that is ideal for efficient phosphoryl transfer in the active site (Wong et al., 2004; Wong et al., 2005; Barkho et al., 2015). Perturbations to the regulatory domains by mutation or ligand binding cause coordinated conformational changes to the N-lobe of the catalytic domain and the active site, resulting in changes in the catalytic activity of Csk (Wong et al., 2005; Barkho et al., 2013). A recent study demonstrated that the α-helix C is an essential region for Csk conformational transition and domain coordination (Dias et al., 2022). Applying a Cα structure-based model *in silico*, they modeled reversible transitions between active and inactive forms as fluctuations between two energetic basins. Their analysis revealed that a conformational change in the α*-*helix C is required for a complete conformational transition. Restrictions in the α*-*helix C region resulted in global conformational changes. Introduction of restrictions in some α*-*helix C dihedral angles, deletions of SH3 and SH2−SH3 domains, and construction of an inhibitor mimetic potential negatively impacted the conformational transitions and, in some cases, vanishing them. These results are consistent with a previous mutagenic study that identified the α-helix C as a key motif of the “on switch” in both Csk and Src (Huang et al., 2009). Differences between Csk and Chk.

Csk and Chk share a 54% amino acid sequence identity, and both have been confirmed to be able to phosphorylate and inactivate Src family kinases (Sun et al., 1998; Ayrapetov et al., 2003). However, they diverged in the context of cellular signaling in two major ways. The first lies in the different specificity of Csk and Chk SH2 domains. The SH2 domain of Chk binds to numerous pTyr-containing receptor kinases, such as ErbB2 (Zrihan-Licht et al., 1997; Zrihan-Licht et al., 1998; Bougeret et al., 2001; Fu et al., 2006), c-Kit (Price et al., 1997), TrkA (Yamashita et al., 1999), and RAFTK (McShan et al., 2002) in numerous cancer cell types and regulate their proliferation, while the Csk SH2 domain does not bind to these receptors. Such SH2 domain-mediated interactions would enable Chk and Csk to participate in regulating different signaling pathways. Binding affinity and specificity analysis confirmed that the Chk SH2 domain is more similar to the Src SH2 domain in binding specificity than the Csk SH2 domain. Mutagenic dissection revealed that this functional diversity in the Csk *versus* Chk SH2 domains is mainly due to one critical residue substitution, Glu^127^ in Csk and Ile^167^ in Chk (Ayrapetov et al., 2005). This diversity offers an interesting example of how a minor change in primary structures can critically alter the function of otherwise very similar proteins.

Another key difference between Csk and Chk is that only Chk can non-enzymatically inhibit the catalytic activity of Src family kinases (Chong et al., 2004; Chong et al., 2006), while Csk is more efficient at enzymatically inactivating Src family kinases by phosphorylation (Advani et al., 2017). Early difficulty in purifying the Chk enzyme from recombinant sources limited the enzymatic characterization of Chk (Ayrapetov et al., 2003). Developing a procedure for expressing and purifying Chk (Chan et al., 2010) enabled detailed comparative studies of Chk and Csk function. Cheng and others revealed that while the interaction between Csk and Src family kinases is transient and catalytic, the binding between Chk and Src family kinases is tight and stable. The tight binding alone inhibits SFKs without phosphorylating them on the C-terminal tail Tyr. This non-catalytic inhibitory binding is a novel mechanism employed by Chk to inhibit SFKs (Chong et al., 2004; Chong et al., 2006; Ia et al., 2010; Advani et al., 2017). This topic has been previously reviewed (Chong et al., 2005a; Chong et al., 2005b; Ia et al., 2011). The non-catalytic inhibition of SFKs by Chk may also contribute to the broad biological differences between Chk and Csk in cancer signaling (Zhu et al., 2008; Chüeh et al., 2021). The kinase domain of Chk contains the structural determinants for the tight binding and non-catalytic inhibition of SFKs. However, the residues equivalent to those for Csk-Src substrate recognition are not involved in the Chk-SFK inhibition (Advani et al., 2017). The exact structural basis of Chk interaction with and inhibition of Src family kinases has yet to be determined.

## 7 Csk regulation by phosphorylation and SUMOylation

It was recently discovered that Csk is also regulated by Tyr phosphorylation *via* a two-tiered mechanism. T-cell activation is dependent on the coordinated activation of Lck, which is regulated by Csk. Csk is phosphorylated by the Activated CDC42 kinase 1 (ACK1) on Tyr18 (Sridaran et al., 2022). The phosphorylation activates Csk kinase activity and promotes Csk polyubiquitination and proteasomal degradation. This regulatory mechanism plays an important role in T-cell activation in immune response. Pharmacological manipulation of this regulatory system by ACK1 inhibitors can help overcome the resistance to checkpoint inhibitors by prostate cancer. The full implications of this regulatory mechanism in Csk regulation of SFKs and checkpoint blockade therapy are still to be investigated.

Csk is also regulated by cAMP-dependent protein kinase (PKA), even though the effect of PKA phosphorylation on Csk is controversial. One study reported that PKA phosphorylates Csk *in vitro*, which inactivates Csk kinase activity (Sun et al., 1997), while others found PKA phosphorylates Csk on Ser364 and activates Csk (Vang et al., 2001; Yaqub et al., 2003). The phosphorylation does not require the regulatory domains of Csk, but the SH3 domain appears to facilitate the activation (Yaqub et al., 2003). The structural basis for either the inactivation or activation has not been elucidated. The PKA-Csk-Lck/Fyn regulatory pathway plays an important role in maintaining T-cell homeostasis (Mosenden & Taskén, 2011).

Csk is also post-translationally modified by small ubiquitin-like modifier, referred to as SUMOylation, on Lys53 both *in vitro* and *in vivo* (Cui et al., 2019), which impairs its binding to CBP and recruitment to the lipid rafts. Thus, SUMOylation decreases Csk function as a negative regulator for SFKs. The mechanism of how SUMOylation affects CBP binding is yet to be determined.

## 8 Redox regulation of PTKs

Redox regulation of PTK activity and protein Tyr phosphorylation-based signaling is widely documented (Corcoran & Cotter, 2013; Truong & Carroll, 2013). Reactive oxygen species are products of oxidative stress and also mediators of the cellular communication (Zhang et al., 2016). Understanding how PTKs respond to redox signaling is important for elucidating the complex influences of reactive oxygen species on global cellular regulation. Csk and Src have been used for investigating the mechanisms of the redox regulation of PTK function. In the crystal structure of Csk, there is a novel disulfide bond between Cys122 and Cys164 (Ogawa et al., 2002). Mills et al. showed that dialysis overnight in the absence of DTT resulted in a loss of 90% of Csk activity, presumably due to the formation of this disulfide bond (Mills et al., 2007). Reduction of the disulfide bond results in conformational changes to a large number of residues in the SH2 domain and the catalytic domain, especially residues in the active site cleft (Mills et al., 2007). Liu and Cowburn showed that the disulfide bond contributes significantly to the stability of the Csk SH2 domain but slightly weakens the binding to a phosphorylated Tyr ligand (Liu & Cowburn, 2016).

**FIGURE 4:**
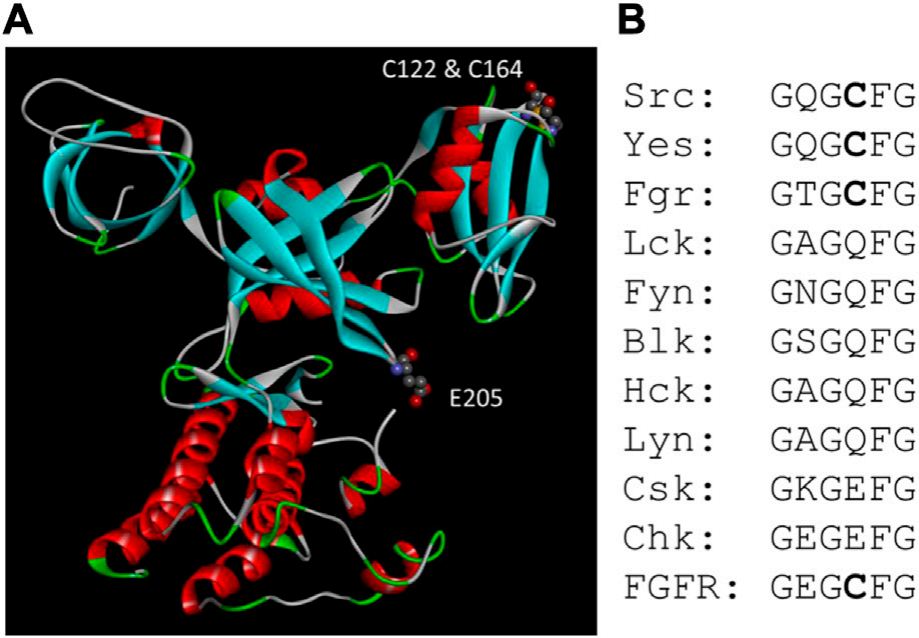
Csk motifs related to redox sensing. **(A)**. Positions of Glu205 and the disulfide bond between Cys122 and Cys164 in Csk structure. **(B)**. Alignment of the Gly-loop sequence in Src, Csk and FGFR family kinases. All four members of the FGFR kinases have the same sequence in the Gly-loop.

Kemble and Sun found that reducing condition has only a minor effect on Csk activity, but it stimulates Src kinase activity approximately 13-fold (Kemble & Sun, 2009). Ala scanning of all Cys residue in Src identified Cys277 located in the Gly-loop of the catalytic domain to be responsible for the redox sensing. When Src is oxidized, it forms an inter-molecular disulfide bond using two Cys277 residues. The Cys residue was conserved in three Src family kinases and four FGFR kinases. The homologous residue in FGFR1 also renders FGFR1 sensitive to redox regulation. Both Cys277 in Src and the equivalent Cys residue in FGFR are located in the highly conserved Gly-loop with the consensus sequence of GXGZXG, where Z is the Cys residue. It has been shown that the Gly-loop is a critical motif for kinase catalysis (Hemmer et al., 1997), and it is understandable that a disulfide bond between the Gly-loop of two PTK molecules would severely impair the kinase function (Corcoran & Cotter, 2013). The equivalent residue in Csk is Glu205. When Glu205 is mutated to a Cys, Csk also becomes sensitive to reducing agents. The position of Glu205 in Csk and the disulfide bond between Cys122 and Cys164 are shown in Figure 4.

While the above studies reveal the potential redox sensing mechanisms embedded in the PTK architecture, caution must be exercised in interpreting *in vitro* results of redox regulation (Sun & Kemble, 2009). The redox state of a given Cys residue is extremely sensitive to its environment. Thus, the redox state of an isolated enzyme is determined by the redox condition of the buffer rather than the environment from which it is isolated. While it is relatively straightforward to determine how an enzyme responds to redox manipulations *in vitro*, it is much more challenging to assess the redox state of an enzyme in the cell under different redox conditions that may induce oxidative stress. This is likely the root cause of conflicting reports of how Src family kinases respond to redox regulation (Giannoni et al., 2010; Dustin et al., 2020).

## 9 Concluding remarks

Protein tyrosine kinases are a large family of enzymes sharing a common catalytic core architecture. Each PTK contains unique structural domains, motifs, or residues that render each PTK unique in its catalytic or regulatory properties. These catalytic and regulatory properties enable each PTK to fit into various regulatory pathways that collectively become the cellular regulatory network. Dissecting the structure-function relationships leads to a better understanding of how PTKs work as individual enzymes and how they carry out their regulatory functions in the cells. Such understanding leads to ways of manipulating cell signaling for therapeutic or investigative purposes. Csk/Chk and the Src family kinases they regulate constitute an important signaling module in mammalian cell regulation and an excellent model system for dissecting regulatory and catalytic structure-function relationships for PTKs. These studies made an important contribution to our current understanding of Tyr phosphorylation as a global regulatory mechanism and a key target for anti-cancer therapeutic development.

## Abbreviations

CBP, Csk binding protein; Chk, Csk homologous kinase; Csk, c-Src, cellular Src; C-terminal Src kinase; FGFR, fibroblast growth factor receptor; IRFs, interferon regulatory transcription factors; PTK, protein tyrosine kinase; SFKs, Src family kinases; SH2 domain, Src homology domain 2; SH3 domain, Src homology domain 3; v-Src, viral Src.
